# An Overview of the Applications of Nanomaterials and Nanodevices in the Food Industry

**DOI:** 10.3390/foods9020148

**Published:** 2020-02-03

**Authors:** Mehwish Shafiq, Sumaira Anjum, Christophe Hano, Iram Anjum, Bilal Haider Abbasi

**Affiliations:** 1 Department of Biotechnology, Kinnaird College for Women, Lahore 54000, Pakistan; mehwishshafiq091@gmail.com (M.S.); iram.anjum@kinnaird.edu.pk (I.A.); 2 Laboratoire de Biologie des Ligneux et des Grandes Cultures, INRA USC1328/Université d’Orléans, 28000 Chartres, France; christophe.hano@univ-orleans.fr; 3 Department of Biotechnology, Quaid-i-Azam University, Islamabad 45320, Pakistan

**Keywords:** food industry, nanomaterials, nanosensors, nanocapsules, food safety, food packaging

## Abstract

The efficient progress in nanotechnology has transformed many aspects of food science and the food industry with enhanced investment and market share. Recent advances in nanomaterials and nanodevices such as nanosensors, nano-emulsions, nanopesticides or nanocapsules are intended to bring about innovative applications in the food industry. In this review, the current applications of nanotechnology for packaging, processing, and the enhancement of the nutritional value and shelf life of foods are targeted. In addition, the functionality and applicability of food-related nanotechnologies are also highlighted and critically discussed in order to provide an insight into the development and evaluation of the safety of nanotechnology in the food industry.

## 1. Introduction

Nanotechnology offers attractive opportunities in the food industry such as for food safety and quality control as well as the production of new food additives/supplements and other flavors [1]. In the food industry, nanotechnology can also be used for the production of packages with enhanced thermal and/or mechanical properties and safety. Indeed, nanosensors embedded in food packaging systems are used to alert consumers when foods have expired. Nanotechnology can also be used to make healthier foods [2]. The variety of nanostructures with diverse properties makes them suitable for addition to foods as well as in packaging products that enhance the nutritional quality of foods [3]. Out of 633 available nanomaterials, 55 are exploited in agriculture and food sciences [4]. A recent report pointed out that food products linked to nanotechnology will account for around 50% of total food products in 2020 [5].

**Figure 1:**
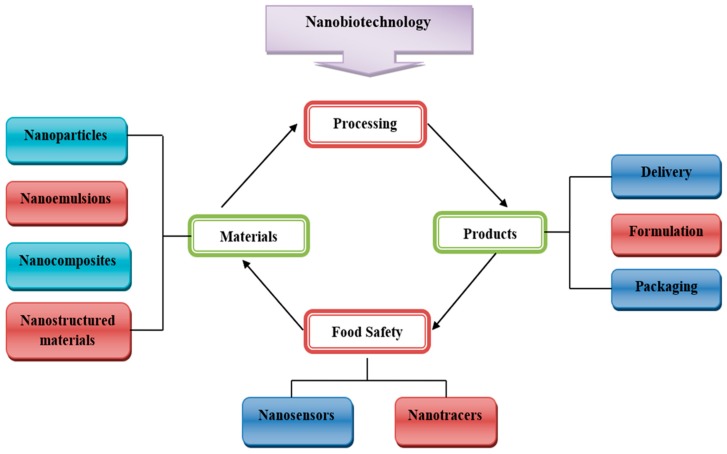
Role of nanotechnology in various sectors of the food industry.

Undoubtedly, nanotechnology is revolutionizing the food industry. Most of the reported applications of nanostructures in food include (i) the improvement of food quality, (ii) bioactive fortification, (iii) controlled release of bioactive compounds using nanocarrier encapsulation, (iv) modification of food structures and textures, and (v) the detection and neutralization of biochemical, microbiological and chemical alterations using intelligent packaging systems (Figure 1) [6]. Different nanomaterials have vast applications in the formation of food products and the improvement of nutritional values. For example, protein nanoparticles are used in the manufacture of food products because protein solubility is helpful in the assembly of protein nanoparticles with preferred functional properties in food substances [7]. 

Currently, the principle applications of nanotechnology in the food sector are mainly related to food nanosensing and nanostructured food ingredients. Nanostructured food ingredients include food formulation or food packaging. For a better assessment of quality and safety, food nanodetection is applied for better results. In food packaging, conventional materials are being replaced by nanopolymers [8]. The modern characteristics of nanotechnology allow the development of new, functional foods in the food industry. New nanomaterial-based engineering approaches for the targeted distribution of nutrients and bioactive compounds in functional foods is another potential application of nanotechnology [9]. The development of nanocapsules, nanocarriers, nanotubes, nanosensors and nanopackages are new opportunities for nanotechnology in the food industry [10]. This review presents recent developments in nanobiotechnology to enhance food safety and/or quality, with a special focus on the toxicity and health issues related to the consumption of these nanoparticles in food. A concise overview from the regulatory point of view is also addressed. 

## 2. Different Aspects and Roles of Nanotechnology in the Food Industry

There is an increasing interest in nanotechnology in the food industry. Several applications have been reported in various dimensions, such as the targeted delivery of nutrients and/or bioactive molecules through nanoencapsulation, the use of biosensors to detect and quantify pathogens and alteration of food composition, or fruit and vegetable preservation by edible films (Table 1) [11]. 

**Figure 2:**
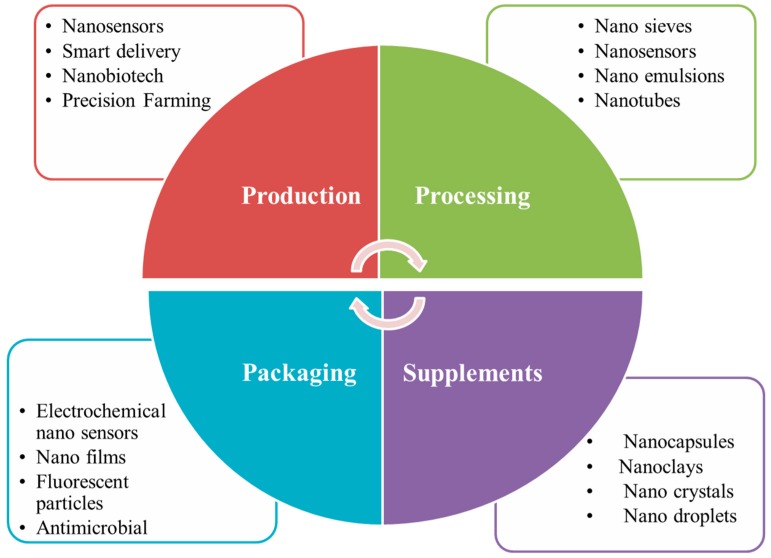
An overview of applications of nanomaterials in the food industry.

Potential applications of nanotechnology in the food chain include food storage, food quality control, food formulation and food packaging (Figure 2) [37]. 

In the food industry, production processes are enhanced by nanotechnology, which provides products with better characteristics as well as with new functionalities.

The use of nanotechnologies can be considered from the production phase, which allows a technical innovation in precision agriculture to improve plant growth, but also the detection of and/or resistance to pests and allelopathy. The nanoencapsulation of conventional fertilizers, pesticides and herbicides allows (i) a slower and longer release of nutrients for more efficient use, allowing the optimal growth of plants; while, for agrochemical products, (ii) it allows safer handling, more efficient usage and a more precise dosage of these compounds with less exposure to the environment, thus guaranteeing the better protection of the environment. In addition, the rapid and early detection of plant diseases based on nanotechnology is also attracting attention. The potential uses and benefits of nanotechnology in precision farming are discussed in detail by Anjum et al. [38] and Duhan et al. [39].

Nanotechnology is also used in food processing and preservation. This application offers a protective barrier to mask different tastes and flavors. Nanotechnology also provides food ingredients as well as water-insoluble food supplements with controlled release and improved dispersibility [40]. The benefits of nanotechnology in food processing include the development of a complete texture for food components, the encapsulation of food additives, the development of new flavors and sensations, and the control of aroma release with increased bioavailability in dietary supplements [41].

Nanotechnology has revolutionized the food industry by designing innovative delivery systems for the production of nano-formulated agrochemicals, improving nutritional values and generating new products through bioactive encapsulation. Nanoencapsulation clearly improves the nutritional values of the product [42]. Indeed, at the nanoscale, the processing of food ingredients allows an improvement of aromas, textures or new tastes, but also enhances nutritional values. Nanoparticles have improved characteristics for product encapsulation as well as a higher release efficiency than conventional encapsulation approaches. Nanomaterial encapsulations protect food products from heat or moisture while controlling the active release of food ingredients and their interaction with other materials in the food matrix. For example, liposomes are delivery vectors for hydrophobic molecules, and nano-emulsions improve oral bioavailability [43]. Nanocochleates are used to deliver nutrients more efficiently to cells without disturbing the taste or color of food [44]. However, self-assembled nanoscale liquid structures are used as vehicles and are called dilated micelles; their size is less than 30 nm. They have been used for the delivery of nutraceuticals and beverages. Potential applications containing lycopene, omega-3 fatty acids, β-carotene and isoflavones are targeted. The American company, Fresh Corporation, has launched an innovative nanoceramic that allows the nano-drying of food, which halves the use of oil in fast food and restaurants because of its immense surface [45]. Therefore, nanoencapsulation allows a precise (bio) availability of the product with a specific rate for the target time [46].

Recently, industrial applications of food nanotechnology have ranged from intelligent packaging to the creation of interactive food. Active packaging is one of the modern terms used for packaging food items in which the packaged foodstuff status changes to improve the sensory quality as well as safety of food products, increasing shelf life by maintaining the quality of food products [47]. Advanced food packaging and nano-based systems minimize food losses due to different microbial infections. Different nanoparticles such as titanium dioxide, magnesium oxide, zinc oxide, silver nanoparticles, carbon nanotubes, fullerene derivatives and zerovalent iron have shown great impacts as antimicrobial agents [48].

Furthermore, the use of specific nanomaterials to detect/eliminate harmful chemicals/pathogens in food products has recently been developed. In advanced food packaging, an integrated electronic tongue contains a collection of nanosensors that are particularly sensitive to the gases released from food waste; this gives a clear and visible signal indicating whether the food is fresh or not, using a sensor strip that changes color [49]. For the rapid detection of food-borne pathogens, magnetic oxide nanoparticles are also used in the food industry [50]. Nanosensors detect microorganisms, toxic substances and contaminants present in different foodstuffs because of their high detection capacity, which is useful for the safety of food products [51].

The applications of nanotechnology in the food industry promote innovations in different dimensions of food items, including texture, taste, sensory attributes, the color strength of products, and the stability/shelf life of products [52]. 

### 2.1. Applications as Food Additives and Food Ingredients

Basically, the ingested food components are carbohydrates, proteins and lipids. However, a common factor between them is the digestion of their components, which occurs at the nanoscale [53]. On this basis, it could be argued that food processing at the nanoscale would only increase the efficiency or speed of digestion, bioavailability, metabolism and absorption in the human body [54]. Obviously, within the food market, there are supplements available that have tri- and di-peptides and are thus further voluntarily digestible. On the contrary, it might be claimed that the processing of substances at this scale frequently changes their properties, rather than processing at the nanoscale level. This important issue needs additional research as it drives significant regulatory questions about modifications in properties [55].

### 2.2. Nanomaterial in Food Processing

**Figure 3:**
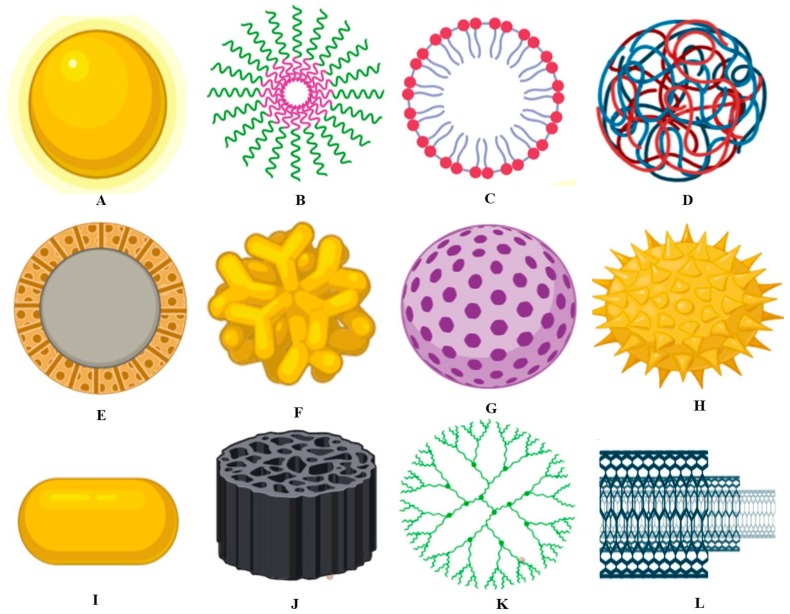
Graphical representation of different types of nanomaterials used in the food industry. (**A**) Metallic nanoparticles, (**B**) Polymeric micelle, (**C**) Liposomes nanoparticles, (**D**) Polymeric nanoparticles, (**E**) Solid-core mesoporous nanoparticles, (**F**) Branched gold nanoparticles, (**G**) Mesoporous nanoparticles, (**H**) Surface functionalized nanoparticles, (**I**) Nanorod, (**J**) Porous silica nanoparticles, (**K**) Dendrimer, (**L**) Carbon nanotubes.

Some food ingredients that work on the nanoscale or are termed as nanostructured have different properties as they improve texture, consistency and taste, etc. Various types of food nanotechnology have been involved in improving shelf life [56]. Today, nanocarriers are used in the same way as delivery structures for different flavors of food in food yields without manipulating the morphology (Figure 3). Particle sizes might openly influence the supply of bioactive complex to numerous sites inside the body; for example, it was observed that the submicron particles can only work on the nanoscale [57]. A perfect delivery method is comprised of the following:

- The ability to bring energetic material specifically to the target position;
- Confirming the accessibility of the target period along with a definite rate;
- Efficiency at keeping compounds that are active at appropriate levels for extended phases of time (storage state). Nanotechnology is functional in the development of emulsions, encapsulations, simple solutions, the association of colloids biopolymer matrices, and well-organized delivery organizations with the above-mentioned abilities [58].

Nanoparticles have enhanced capabilities such as release efficiency and encapsulation properties when compared with traditional encapsulation methods. Nanoencapsulation covers tastes and the odor control connections of potent ingredients by means of the food matrix, release of active compounds, confirmation of the availability of the target time, and protection from different sources of contamination such as heat, moisture, biological and chemical degradation [59] during storage, processing and utilization.

These nanoparticles also show compatibility when compared with other compounds in the system [60]. Furthermore, these delivery classifications retain the capability to enter into tissues owing to their small size and thus permit the well-organized distribution of active complexes to mark sites in body [61]. Various artificial and ordinary polymer-based encapsulation delivery classifications have been explained for better-quality bioavailability as well as the protection of active components of food. Moreover, the significance of nanotechnology such as in food processing can be estimated by seeing its role in the enhancement of food items [62].

### 2.3. Nanomaterials in Improving the Texture, Appearance, Taste and Nutritional Values of Food

Nanotechnology provides several opportunities for improvements in food value and taste. Nanoencapsulation methods are recycled largely for progressive taste release along with maintaining a culinary balance [63]. Nanoencapsulation is used for extremely reactive plant pigments that are unstable, such as anthocyanins. Photostability and thermal stability can be improved by encapsulating such molecules as cyanidin-3-O-glucoside inside the middle cavity of recombinant soybean kernel. H-2 subunit ferritin (rH-2) enhances photostability and thermal stability. Rutin is considered to be a dietary flavonoid with abundant significant pharmacological properties, but owing to its reduced solubility, its utilization in the food industry is less common. The encapsulation of ferritin nanocages improved the thermal, UV radiation and firmness properties of ferritin-confined rutin as related to unattached rutin [64]. The usage of nano-emulsions to create lipid-soluble compounds that are bioactive is much more common, as it has also found applications in improving bioavailability and water-dispersion [65].

The majority of bioactive complexes, such as carbohydrates, lipids, vitamins and proteins, are fragile in a highly acidic environment along with enzyme action in the duodenum and stomach. Bioactive multifarious encapsulation not only allows them to fight that difficult situation but permits them to adjust willingly into food goods [66]. This phenomenon is quite difficult to attain by the usage of non-capsulated bioactive compounds that have less solubility. To enhance the delivery of vitamins, fragile micronutrients, and medicines, currently, small edible capsules coated with nanoparticles are used in daily foods to provide beneficial health effects [67].

### 2.4. Nanomaterials in Food Packaging

**Figure 4:**
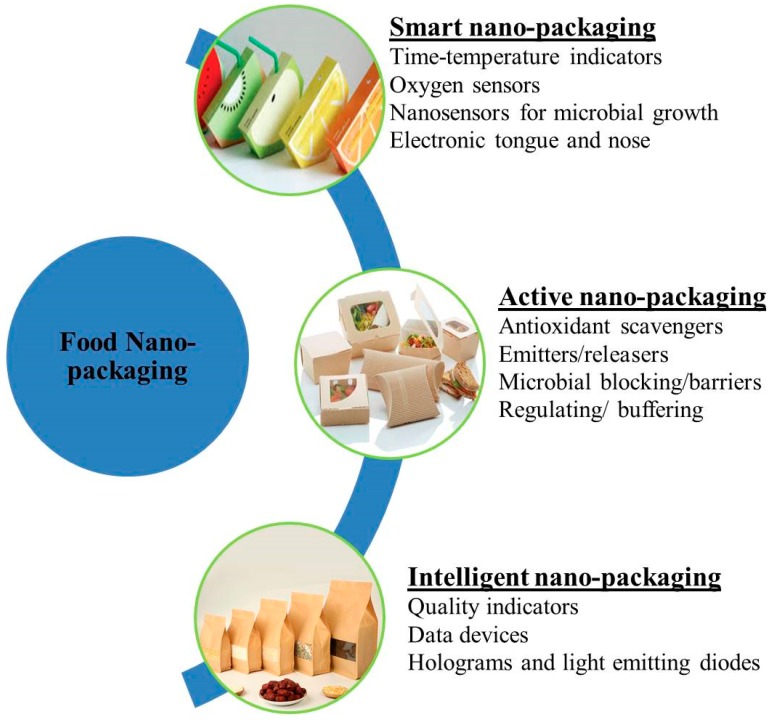
Classification of food nanopackaging.

Packaging plays an essential role in the modern food industry. However, the most valuable process in the preservation of food products is the improvement in quality from production to consumption [68]. Nanotechnology applications in the food packaging field offer novel promises for improvements in the efficiency of foodstuff packages (Figure 4). Many nanoparticles are produced and used in the industry due to their encapsulation ability in active compounds and enhancement of functionality, stability, and bioavailability [69]. Nanofillers are also used in food packaging due to their desired functions as well as applications in the packaging of food. In biosensors, nanofillers not only shelter food from environmental factors but also integrate properties in packaging materials and permit numerous potential opportunities in the food packaging industry (Figure 4) [70]. 

Zinc oxide is a nanocomposite material which is used in the active packaging of food materials as it has an antioxidant effect, which is important for the food industry. The main degradation reaction is the oxidation that occurs in food, limiting its preservation. Antioxidants are incorporated in the packaging material in the active packaging approach that maintains the preservation of food products [71]. Not only are nanoparticles used in the antimicrobial packaging of food items, but nanocomposites along with nanolaminates also extensively used for the packaging of food. They are used to protect food from severe temperatures and mechanical stunning and to extend shelf life. The incorporation of nanoparticles into the packaging of food materials offers a longer shelf life and better quality of food [72].

The smart packaging of food constitutes active and intelligent packaging. Active packaging generally enhances the shelf life, and maintains and improves the packaged food. Bioactive packaging provides a positive impact for the health of consumers with the production of packaged foods which are good for health [73]. A wide range of nanoparticles have antimicrobial effects and have attracted a great deal of attention in a series of applications in food packaging. Metal nanoparticles have been incorporated in polymer films of metal nanocomposites in a new generation of nano-food-packaging, which is based on the antimicrobial active packaging of food products; for example, silver nanoparticles are used for food packaging as an active system due to their antimicrobial activity [74]. The most basic commercial applications of nanotechnology are in food packaging in the food industry. The incorporation of nanomaterials in food products improves the packaging properties including the flexibility as well as gas barrier properties, which is the most important application of nanotechnology in the food industry [75]. Active packaging includes nanoparticles with antimicrobial and oxygen scavenging activities, smart packaging, and intelligent food packaging, including nanosensors which are intended to sense microbial as well as biochemical alterations and provide signals [76].

The packaging of numerous food items is improved through nanotechnology. New nanopackages for foodstuff acquire exclusive properties; i.e., they are capable of destroying microbes that are present in food substances [77]. Nano materials used for the wrapping of food products enhance the existence of foodstuff products without causing any unwanted modification within the features of products [33]. Nanoparticles are also used for the nanopackaging of food substances. This application of nanotechnology give solutions intended for the packaging of food through modification in the penetration activities of foils, increasing the mechanical, chemical and microbial obstacle effects as well as resistant to heat [78].

Nanotechnology decreases environmental pollution by the production of biodegradable packaging. A silicate layer is a nanostructure that is used in the packaging of food products in the food industry. Improvements in the sensor technology present in the smart packaging of food material provide information on the quality and safety as well as the half-life of materials [79]. Nanocomposites are also used in the packaging of food products. They have enhanced properties to resist thermal stress in food processing, as well as the transportation and storage of food products. Currently, nanocomposites are used in bottles of beer, enhancing their shelf life by up to 6 months. Carbon-based graphene nanoplates are resistant to heat and have potential applications in the packaging of food products in the food industry [80].

A technique is used by researchers known as sonochemical coating, which is a versatile and simple technique utilized for the creation of coating materials using ultrasonication [81]. The coating has been verified to be fruitful against numerous bacterial strains (Gram positive *E. coli* or Gram negative *S. aureus* bacteria). The method has been verified to advance the production of materials which can help food to be conserved for an extended duration. 

Silver nanoparticles are considered to be important particles for material packaging and are used for longer period of time. For example, substances that are covered by such particles are prevented from contamination. Many researchers have stated that silver nanoparticles have importance in research involving food packaging and in the food preservation industry, although only some methods of silver nanoparticles are certified by the EFSA (European Food Safety Authority) to be able to be recycled in food preservation and packaging [82]. For everyday applications, zinc oxide is termed as a safe material, certified by FDA, and is considered as a food additive [83]. Nanotechnology produces many antimicrobial agents with novel properties such as zinc oxide, magnesium oxide, nickel oxide and silver nanoparticles. These nanoparticles have shown potential applications and antimicrobial properties at the nanoscale. These nanoparticles are incorporated in matrices of polymers to provide good properties such as antimicrobial activity and enhance the properties of packaging [84].

### 2.5. Nanomaterials in the Preservation of Food

Different nanoparticles are used in the preservation of food. For example, zinc oxide exhibits antimicrobial activities along with inherent functions in the preservation of food [29]. Nanotechnology is helpful in the preservation and maintenance of the quality of food products as well as improving the following characteristics: (1) product appearance, (2) the function of products, and (3) the nutritional and sensory attributes of products. Metallic nanoparticles such as silver nanoparticles are used not only for antimicrobial preservatives but have encouraging roles in preserving food products. Titanium dioxide is a white color enhancer in food products; for example, it is added in milk, cheeses, and other dairy products to enhance the white color appearance [85]. The bioactive component becomes degraded and inactivated in functional foods, and the nanoencapsulation of bioactive constituents increases the shelf-life of food goods through a reduction in the process of degradation; otherwise, degradations are stopped until the product is brought to its intended place. Furthermore, edible nanocoatings for numerous foodstuff substances might produce an obstacle to gas interchange as well as creating flavors, colors, antioxidants, and anti-browning mediators as well as enzymes which also increase the shelf life of artificial foodstuffs. By changing the properties of the interfacial layer with the help of the encapsulation of nanoparticles, it is possible to decrease degradation [86].

## 3. Nanosensors as Emerging Devices in the Food Industry

Nanosensors are bioanalytical devices that are developed by using various nanostructured materials and biological receptors in an integrated system design. Nanosensors play an important role in the food industry and have attracted much attention in recent times due to their quick detection capacity, integrity and cost-effectiveness [87]. Nanosensors have the potential to be integrated with an array of analytes due to their high sensitivity and specificity. These devices have a high surface-to-volume ratio and excellent optical and electric properties due to conjugation with various types of nanomaterials such as carbon nanotubes, nanoparticles (metallic, non-metallic and metal oxide), semiconductor nanoparticles, nanorods, nanowires, nanobiofilms, nanofibers, and quantum dots [88,89]. Currently, nanosensors are being used in the detection of food-borne pathogens, adulterants, toxins, chemicals and pesticides which are present in different foodstuffs [90]. They also used to monitor the freshness of food and food packaging integrity [77]. Different types of techniques/methodologies such as cyclic voltammetry, surface plasmon resonance, differential pulse voltammetry, interdigitated array microelectrode-based impedance analysis, amperometry, flow injection analysis and bioluminescence are employed as nanobiosensing tools to rapidly and accurately detect different pathogens, toxins and adulterants present in foods [91,92,93,94,95]. The potential applications of nanosensors in various sectors of the food industry are summarized in Table 2. 

### 3.1. Nanosensors in the Detection of Toxins

The micro fluidic sensor is a type of nanosensor based on microfluidics along with liposomes, providing advantages in the detection of toxic substances in aqueous samples even within the micro liter range [96]. Electrochemical sensors and biosensors based on novel nanomaterials such as carbon nanotubes (single and multi-walled), metallic nanoparticles (silver, gold, platinum, copper and zinc), and superparamagnetic nanoparticles are currently used in the detection of various toxins present in foodstuffs [90,97]. A group of toxic and carcinogenic compounds known as aflatoxins have been found in food contaminated with *Aspergillus flavus* and *Aspergillus parasiticus*. Gold nanoparticles functionalized with anti-aflatoxin antibodies have been used for the detection of aflatoxin B1. Likewise, superparamagnetic beads containing anti-aflatoxin M1 antibodies and gold nanoprobes have also been used for the detection of aflatoxin M1 in milk samples [98,99,100,101]. Similarly, gold nanoparticles acting as nanoprobes in enzyme-linked immunosorbent and immune-chromatographic assays have been used for the detection of botulinum neurotoxin type B and brevetoxins present in processed foods [102,103]. Contaminated seafood generally contains marine toxin, namely palytoxin, which has also been detected by using carbon nanotube-based electrochemiluminescent sensors [104,105].

### 3.2. Nanosensors in the Detection of Food Pathogens

Foodborne pathogen detection in food materials is mainly achieved by identifying the bacterial genetic material or whole bacterial cell. The control of pathogens by using these conventional microbiological techniques is very reliable, but at the same time, it is very complicated [106]. Nanotechnology allows the implementation of low-cost nanosensors in the packaging of food to detect different pathogenic microorganisms present in various food products [107]. The nanobioluminescent spray, one of the most efficient nanobiosensors, produces a visual glow for the easy detection of pathogen strains in different food products. This spray is made up of different magnetic nanoparticles which react with pathogens present in food substances and produce a visual color that can be easily detected [108]. Magnetic iron oxide nanoparticles have been used to isolate the DNA of the milk pathogenic bacterium *Listeria monocytogenes* [109]. Various nanosensors integrated with different types of nanomaterials have been documented for detecting pathogenic bacteria in standard bacterial culture samples as well as complex food samples (Table 2).

Surface-enhanced Raman spectroscopy coupled with silver nanosensors is one of the most efficient techniques in detecting pathogenic bacteria [110]. Besides silver, nanosensors integrated with other nanomaterials such as nanorods, nanocolloids, graphene oxide, carbon nanotubes, plamonic gold and magnetic beads are also routinely used for the detection of foodborne pathogens [111,112,113,114]. In recent years, an array-based immunosorbent assay coupled with liposomal nanovesicles has gained popularity due to its efficacy and specificity in detecting *E. coli*, *L. monocytogenes*, and *Salmonella* spp. [115,116,117]. Further, silicon-based nanosensors coupled with proteins that vibrate at different frequencies depending on their biomass have been used for pathogen detection in various mixed liquid food systems [118]. In recent years, various new nanoparticle-based detection platforms such as lateral-flow immune test strips with palladium nanoparticles against *Klebsiella* and field effect transistors with graphene-based nanoparticles against *E. coli* have been developed [119,120].

### 3.3. Nanosensors in Sensing Chemicals and Pesticides in Food

Nanosensors composed of different nanomaterials have been used for the detection of pesticides, fertilizers and other toxic chemicals present in various foodstuffs (Table 2). Colorimetric and fluorometric nanosensors conjugated with gold nanoparticles have been used for the detection of organophosphorus and carbamate pesticides [121]. Core-shell quantum dots made up of cadmium selenide and zinc sulfide have been explored for paraoxon sensing [122]. Potentiometer sensors based on silica nanocomposites and multi-walled carbon nanotubes have been reported for the detection of toxic cadium ions [123]. Similarly, voltametric nanosensors based on nanocomposite biofilms (gold/zirconium dioxide) and fluorescent nanosensors (conjugated with gold nanoparticles) have been developed for the detection of parathion and melamine pesticides, respectively [124,125].

Food dyes and preservatives are also toxic when used above permissible limits. Ionic-liquid nanocomposites modified with multi-walled carbon nanotubes have been employed for the detection of food dyes such as sudan-I (a carcinogenic red dye used as an adulterant in chili powder tomato ketchup, strawberry and chili sauce), sunset yellow, and tartrazine [109,126]. Likewise, nanocomposites in conjugation with zinc oxide nanoparticles and carbon nanotubes have been reported for the simultaneous detection of bisphenol A (a toxic organic compound released from plastic containers) and sudan I [92,127].

### 3.4. Nanosensors in Sensing the Quality of Key Food Ingredients

Vitamins and other key food ingredients such as antioxidant compounds are necessary for the normal function of various metabolic pathways in our body, and their deficiency can lead to serious health issues such as anemia, cardiovascular diseases, and carcinogenesis. These key food ingredients are easily degraded in different processed foods. To avoid their degradation, different types of nanosensors conjugated with various nanomaterials have been reported to date (Table 2). Ionic liquid nanocomposites based on carbon nanotubes and nickel oxide nanoparticles have been reported for the detection of ascorbic acid and folic acid in various fruit juices, wheat flour and milk samples [128,129,130]. The level of succinic acid, citric acid, L-malic acid, fructose, D-sorbitol, sucrose, glucose, hydrogen peroxide and L-glutamic acid in stored food products is used as an indicator of food quality [131,132]. Different types of nanosensors in conjugation with silver, zirconium dioxide, iron, nickel–platinum, chitosan, gold, tin dioxide, prussian blue–gold and cuprous oxide nanoparticles have been used to monitor the quality of the above-mentioned food ingredients in various food components [115,133,134,135,136]. 

## 4. Nanomaterials and Devices in Food Safety

In the food industry, the safety of food products is an important issue. According to a recent survey, more than 45% of processed and packaged food items are prone to degradation and contamination [185]. Nanotechnology has played a positive role in solving various issues related to the quality and safety of food products [171]. Currently, different types of nanomaterials and nanodevices such as polymeric nanoparticles, liposomic nanovesicles, nanoloaded emulsions and temperature–time indicators are used for the improvement of the quality of food by increasing shelf life, sensing freshness, and detecting chemical, heavy metal, and allergen contamination in food items [186,187,188]. Different nanosensors and nanotracers conjugated with various nanomaterials such as gold nanoparticles, silicon nanorods, magnetic beads, quantum dost, single-walled and multi-walled carbon nanotubes, immunomagnetic liposomes, aptamer conjugated gold, and palladium nanoparticles are also highly efficient in detecting various contaminants and degradants that influence the quality of food [126,189,190]. Additionally, the newly developed radio frequency identification technique (RFID) is found to be well suited for numerous operations in food engineering and supply chain supervision due to its speed and effectiveness [191]. RFID technology may also deliver safety and security improvements for food corporations by tracing the source of contaminants in various food products [192]. The role which various nanomaterials and devices play in ensuring food quality and food safety are briefly discussed in this review. 

### 4.1. Nanobarcodes for Product Authenticity

Two-dimensional barcodes are used globally for the visual authentication of products; however, they can be easily altered, falsified, and damaged. To solve these issues, nanoparticle and nanodisk-based unique invisible barcodes have been developed in recent years to confirm the authenticity of various food products [193,194]. The nanodisk barcode can be scanned with a Raman microscope, which could be in the form of a linear gold nanodisk array, silver–gold heterodimer nanodisk, or silver nanodisk codes [195]. Nanodisks can be functionalized and amalgamated with metal composites to improve their properties further. Fluorescent-based barcode nanorods, invisible nanobarcodes, and fluorescent DNA dendrimer nanobarcodes have been reported for product labelling and pathogen detection in food and biological samples [196,197,198,199]. Henceforth, nanotechnology will find use in developing efficient nanobarcode systems to ensure food quality and safety.

### 4.2. Nanomaterials for Protection from Allergens

Nanotechnology also finds application in controlling and managing various food allergens [200,201,202]. Conventional adjuvants such as aluminum hydroxide (alum) present several side effects including indurations, swelling, erythema, granulomas, and cutaneous nodules at the site of injection [203]. In contrast to conventional adjuvants, nanomaterials such as polymer or protamine-based nanoparticles with Toll-like receptor 9 (TLR-9) ligand cytosine phosphate guanine-oligodeoxynucleotides are used as adjuvants and delivery systems due to their biodegradability, low toxicity, low dosage, reduced allergen exposure to IgE, and efficiency [156,204]. These protamine-based nanoparticle adjuvants also act as a novel carrier system in allergen immunotherapy by counteracting Th2-type immune responses [205]. 

The development of aptamer-based gold nanorod and magnetic nanoparticle fluorescence assay has been reported to detect ochratoxin A in grape juice samples (a food mycotoxin causing allergy) and allergens in different food matrices [206,207]. Moreover, other nanomaterials such as polyanhydride nanoparticles, quantum dots, and dendrimers proved to be efficient oral vehicles for immunotherapy against experimental peanut allergies [208]. Furthermore, the newly synthesized bioinspired nanostructured materials (green synthesized) can be potentially exploited in the food industry to overcome the issues of food allergens due to their low toxicity and cost-effectiveness [209].

### 4.3. Nanomaterials for the Inhibition of Biofilm Formation

Biofilms pose a problem in the food industry, as they are bacterial cells which adhere together very tightly and secrete a polymeric extracellular matrix which is impenetrable [210]. They are formed by the adherence of free-floating microbes on a substrate surface through Van der Waals forces, leading to biofouling, biocorrosion, and interference in food processing [211]. Various types of nanomaterials play a positive role in controlling the biofilm formation in food items, such as filter membranes made up of nanofibers, which have proved to be very efficient in the inhibition of the biofilm formation of many multi-drug resistant bacterial strains [212]. Moreover, the use of different nanoparticles such as silver, nickel oxide, and zinc oxide in anti-bacterial, anti-fungal, anti-biofilm formation has been well documented in many scientific reports [213,214,215]. The fermentation of *Bacillus subtilis* produces high-value products in an industrial setting, but on the other hand, it also hinders the processing of food products through biofilm formation. The efficacy of naked and coated superparamagnetic iron oxide nanoparticles against *B. subtilis* was evaluated by Ranmadugala et al. [216]. Both types of iron oxide nanoparticles significantly reduced the bacterial growth and biofilm formation without affecting the cell viability. Similarly, gold nanoparticles conjugated with chlorhexidine prevented the biofilm formation of *K. pneumonia* and S. aureus [217,218].

## 5. Safety Issues of Nanomaterials in the Food Industry

As is well known, the use of various nanomaterials in the food industry has numerous advantages; however, at the same time, they also pose serious threats to human health, the environment, and other ecosystems due to their cytotoxic effects [219,220,221,222,223,224,225,226,227,228]. Some serious concerns have arisen recently regarding the use of nanomaterials, even those with no toxic element in their composition, but they have an inherent potential risk due to their small size and subcellular interaction with cells [229]. For example, some nanoparticles have the ability to penetrate within skin and cause health problems in humans as well as in animals. Nanoparticles can cause genomic and proteomic changes even in plants and can affect their growth rate [230]. Experimental studies have shown that single and multi-walled carbon nanotubes can induce fibrosis as well as oxidative stress in the lungs of models animals such as mice and rats [231].

To understand the mechanisms of toxicity of different nanomaterials to human health and the environment, we first have to define a clear description of the various exposure routes and entry pathways of nanomaterials from the food industry to human body. We purposely or accidentally consume several processed food items bearing numerous nanomaterials through intraoral, dermal and pulmonary pathways. Oral captivation is the main pathway by which we intake chemicals, water, and nutrients into our bodies. Certainly, numerous nanomaterials are involved in various food items today, and it is supposed that the digestive organs are exposed directly to these nanomaterials every day. Nanoparticles swallowed orally move from the mouth to stomach and then intestines, posing serious health-related risks to human [84,85].

To evaluate the toxicity of nanomaterials, zebrafish are used as a model organism to overcome the challenges including the development and maintenance of cell cultures for animals and human cells. This vertebrate fish has the ability to reproduce rapidly. The U.S. Food and Drug Administration (FDA) and European Union (EU) gave approval for the use of zebrafish for the evaluation of toxicity of nanomaterials [232]. The complete evaluation of various end points related to toxicity specifies that the nanomaterials do not cause developmental defects, mortality or alteration in the behavior of zebrafish. Furthermore, the uptake and biodistribution analysis of nanostructures reveals that they are present in specific locations within body [233]. The recent cytotoxicity evaluation of green synthesized zinc oxide nanoparticles has been carried out in in vitro as well as in vivo, clearly indicating that the green hydrothermally synthesized nanoparticles are more biocompatible and less toxic compared to chemically synthesized nanoparticles [234]. For biosafety and biokinetics assessments of metallic nanoparticles such as gold, silver, and platinum, murine is used as a model organism. This study confirmed the enhanced excretion of nanoparticles through the renal pathway due to their small size. Additionally, the biosafety of all metallic nanoparticles was fully assessed in zebrafish for the evaluation of toxicity within the whole body [235].

The bioaccumulation of nanomaterials derived from either nanopackaging or nanoprocessed items has been confirmed in food and human beings [73]. Therefore, the risk assessment procedures must be strictly followed while processing food items [74,75]. Even with the advent of nanotechnology, the challenges to the development of a healthy and sustainable food industry remain obscure. With the intervention of nanotechnology in the food industry, the public should be educated regarding the possible risks associated with nanomaterials to human health and the environment. Several EU and non-EU countries have designed several regulatory frameworks for dealing with nanomaterials to ensure the safety of nanoproducts in feed, agriculture and food sectors [236]. Additionally, supervisory authorities such as the FDA and Environmental Protection Agency (EPA) have made several amendments to various criteria intended for marketable foodstuffs in terms of health and the quality and safety of products [237]. 

## 6. Conclusions

As the scope of nanobiotechnology has moved forward in recent years, nanomaterial-based devices have become more sensitive and smaller in size. These nanomaterial devices play an important role in the food industry including in the packaging, processing, and preservation of food. Nanomaterials also increase the shelf life of foodstuffs by protecting them from moisture, gases, and lipids. They offer better vehicle systems for the delivery of bioactive compounds. Various nanomaterials and nanosensors also ensure the safety and quality of food. Although advances in nanobiotechnology increase day by day, they also have challenges. The environmental effects and safety issues must be a first priority in research to cope with the hazards associated with the use of nanomaterials and nanodevices in the food industry. In conclusion, nanotechnology, while still in its infancy, has already begun a revolution in the food industry due to advanced nanomaterials and nanodevices. Nanotechnology still has great potential, and new applications are being explored in various areas of the food industry. It is clear that security and safety issues are emerging and will need to be carefully considered and addressed in the future.

## Figures and Tables

**Table 1:** An overview of various applications of nanomaterials in the food industry.

Nanomaterials	Type of Nanomaterials	Applications in Food Industry	References
Nanoparticles	Ag, ZnO, Mg, SiO_2_	Food packaging, oxidation of contaminant, anti-bacterial	[12,13,14]
Nanosieves	Specific nanoparticles	Removal of pathogens or contaminants	[15,16]
Nanocapsules	Bioactive compounds	Increased efficacy and water solubility, local and controlled release	[17,18]
Nano-emulsions	Tweens or spans; gum arabica or modified starch, soy, caseinate	Food encapsulation, food processing, antimicrobial and storage, stability, colorant	[19,20,21]
Nanospheres	Starch nanosphere	Food encapsulation, synthetic adhesives	[22,23,24]
Nanosensors	Aptasensors	Detection of micro-organisms, food deterioration control	[25,26,27]
Nanocochleates	Coiled Nanoparticles	Enhanced nutritional value of food, antioxidant, food protection and stability	[28,29,30]
Nanocomposite	Fe-Cr/Al_2_O_3_ Ni/Al_2_O_3_	Enhanced shelf life of food, food protection and food packaging	[31,32,33]
Nanomicelles	Aquanova, novasol	Liquid carrier, enhanced solubility	[34,35,36]

**Table 2:** Summary of various applications of nanosensors in ensuring food quality and safety.

Nanosensors Based Applications	Nanomaterial Used	Analyte Detected	Method of Detection	References
**Detection of toxins**	Magnetic nanoparticles	Mycotoxin	Immunoassay and enzyme-linked immunosorbent assay	[137]
	Quartz nanopipettes	Zearalenone and HT-2	Ion nanogating and enzyme-linked immunosorbent assay	[138]
	Ionic liquids (gold and graphene oxide), Cerium dioxide and zinc oxide nanoparticles	Ochratoxin-A	Cyclic voltammetry and impedance	[139,140,141]
	Gold nanoparticles	Botulinum neurotoxin type B and brevetoxins	Enzyme linked immunosorbent assay, cyclic voltammetry and immune-chromatographic assays	[102,103]
	Single-walled and multi-walled carbon nanotubes	Palytoxin and Microcystin-LR	Electro-chemiluminescence and immunoassay	[104,105]
	Gold, iron oxide and Superparamagnetic nanoparticles	Aflatoxins B1 and aflatoxin M1	Immunoassay and enzyme-linked immunosorbent assay	[98,99,100,137,142]
**Detection of microbes**	Single-walled carbon nanotubes	*Salmonella infantis* and *E. coli*	Field-effect transistor and fluorescence microscopy	[94,143]
	Core shell nanoparticles (Zinc sulfite coated cadmium selenide)	*E. coli* and *S. typhimurium*	Fluorescence microscopy	[135,144,145]
	Polypyrrole nanowires	*Bacillus globigii*	Linear sweep voltammetry	[146]
	Tris-hexahydrate doped silica nanoparticles	*E. coli*, *S. typhimurium* and *B. cereus*	Spectro-fluorometry and flow cytometry	[143]
	Gold nanoparticles	*E. coli*, *Staphylococcus aureus*, *Vibrio parahaemolyticus*, *Salmonella enterica* and *Salmonella typhi*	Cyclic voltammetry, surface plasmon resonance and differential pulse voltammetry	[147,148,149,150]
	Bismuth nanofilm, iron oxide nanoparticles and peptide nanotubes	*E. coli*, *S. typhimurium* and *L. monocytogenes*	Interdigitated array microelectrode based impedance analysis, cyclic voltammetry, amperometry, flow injection analysis and bioluminescence	[101,124,151,152,153]
	Gold/silicon nanorods	*Salmonella enterica*, Respiratory syncytial virus and *E. coli*		[125,154,155]
	Aptamer conjugated gold nanoparticles	*Salmonella typhimurium*		[156,157]
	Quantum dot	*Salmonella enterica*, *E. coli*, and *Listeria monocytogenes*		[125,158,159,160]
	Magnetic bead and magnetic nanoparticles	*E. coli*, *S. aureus*, and *S. epidermidis*		[150,161]
	Liposome nanoparticle	*Cronobacter sakazakii* and *Salmonella typhimurium*		[162,163]
**Detection of pesticides and chemicals**	Poly(ethylene glycol dimethacrylate-*N* methacryloyl-l-histidine methylester)	Chloramphenicol	Surface plasmon resonance and Ultraviolet–visible spectroscopy	[164]
	Multi-walled carbon nanotubes, iron oxide nanoparticles and graphene	Sudan I	Cyclic voltammetry and high performance liquid chromatography	[109,165,166,167]
	Single-walled carbon nanotubes, multi-walled carbon nanotubes conjugated with silica, platinum and zinc oxide nanoparticles, and ionic liquids of multi-walled carbon nanotubes	Cadmium ions, sunset yellow, Bisphenol A and tartrazine	Field effect transistor and cyclic voltammetry	[92,123,126,127]
	Zinc sulfide–cadmium selenide, liposome, gold, cadmium and selenide zirconium dioxide nanoparticles	Parathion, paraoxon and carbamate pesticides	Square wave voltammetry, photoluminescence, colorimetry, fluorescence based ultraviolet–visible spectroscopy	[121,122,168,169,170]
	Cobalt nitroprusside	Sulfite	Cyclic voltammetry	[171]
	Silver and gold nanoparticles	Melamine	Fluorescence and colorimetric based ultraviolet–visible spectroscopy	[172,173,174,175,176]
**Detection of unstable key food ingredients**	Diphenylalanine peptide nanotubes, multi-walled carbon nanotubes, gold and nickel oxide nanoparticles	Ascorbic acid, acetaminophen, glucose and tryptophan	Amperometry and cyclic voltammetry	[128,136,177,178,179]
	Platinum–cobalt, single-walled, double-walled and mutli-walled carbon nanotubes	Folate and vitamin B9	Cyclic voltammetry	[129,130,180,181]
	Silver, zirconium dioxide, iron, nickel–platinum, chitosan, gold, tin dioxide nanoparticles and prussian blue–gold and cuprous oxide conjugated single-walled carbon nanotubes	Hydrogen peroxide, glucose, fructose, sucrose, glutamic acid and succinic acid	Ultraviolet–visible Spectroscopy, cyclic voltammetry and amperometry	[93,95,133,134]
	Silver–tin dioxide nanoparticles	Ethanol	Adsorption	[182]
	Gold nanoparticles	Caffeic acid, gallic acid catechol and chlorogenic acid	Amperometry and cyclic voltammetry	[183,184]

## References

[1] Dimitrijevic M., Karabasil N., Boskovic M., Teodorovic V., Vasilev D., Djordjevic V., Kilibarda N., Cobanovic N. (2015). Safety aspects of nanotechnology applications in food packaging. Procedia Food Sci..

[2] Gokularaman S., Cruz S.A., Pragalyaashree M., Nishadh A. (2017). Nanotechnology approach in food packaging-review. J. Pharm. Sci. Res..

[3] Aditya A., Chattopadhyay S., Gupta N., Alam S., Veedu A.P., Pal M., Singh A., Santhiya D., Ansari K.M., Ganguli M. (2018). ZnO nanoparticles modified with an amphipathic peptide show improved photoprotection in skin. ACS Appl. Mater. Interfaces.

[4] Belluco S., Gallocchio F., Losasso C., Ricci A. (2018). State of art of nanotechnology applications in the meat chain: A qualitative synthesis. Crit. Rev. Food Sci. Nutr..

[5] Jiménez D., de Miguel-Díez J., Guijarro R., Trujillo-Santos J., Otero R., Barba R., Muriel A., Meyer G., Yusen R.D., Monreal M. (2016). Trends in the management and outcomes of acute pulmonary embolism: Analysis from the RIETE registry. J. Am. Coll. Cardiol..

[6] Steinvil A., Zhang Y.-J., Lee S.Y., Pang S., Waksman R., Chen S.-L., Garcia-Garcia H.M. (2016). Intravascular ultrasound-guided drug-eluting stent implantation: An updated meta-analysis of randomized control trials and observational studies. Int. J. Cardiol..

[7] Xu W. (2019). Three-Dimensional Printing of Wood Derived Biopolymers Towards Biomedical Applications. Ph.D. Thesis.

[8] Rashidi L., Khosravi-Darani K. (2011). The applications of nanotechnology in food industry. Crit. Rev. Food Sci. Nutr..

[9] Chang P.-S., Umakoshi H., Kim H. (2019). Nanotechnology for food engineering: Biomembrane and nanocarriers. J. Chem..

[10] Ummi A.S., Siddiquee S. (2019). Nanotechnology applications in food: Opportunities and challenges in food industry. Nanotechnology: Applications in Energy, Drug and Food.

[11] Durán N., Marcato P.D. (2013). Nanobiotechnology perspectives. Role of nanotechnology in the food industry: A review. Int. J. Food Sci. Technol..

[12] Shi L.-E., Li Z.-H., Zheng W., Zhao Y.-F., Jin Y.-F., Tang Z.-X. (2014). Synthesis, antibacterial activity, antibacterial mechanism and food applications of ZnO nanoparticles: A review. Food Addit. Contam. Part A.

[13] Chernousova S., Epple M. (2013). Silver as antibacterial agent: Ion, nanoparticle, and metal. Angew. Chem. Int. Ed..

[14] Hoseinnejad M., Jafari S.M., Katouzian I. (2018). Inorganic and metal nanoparticles and their antimicrobial activity in food packaging applications. Crit. Rev. Microbiol..

[15] Maguire-Boyle S.J., Liga M.V., Li Q., Barron A.R. (2012). Alumoxane/Ferroxane nanoparticles for the removal of viral pathogens: The importance of surface functionality to nanoparticle activity. Nanoscale.

[16] Smith S.C., Rodrigues D.F. (2015). Carbon-Based nanomaterials for removal of chemical and biological contaminants from water: A review of mechanisms and applications. Carbon.

[17] Cosco D., Paolino D., De Angelis F., Cilurzo F., Celia C., Di Marzio L., Russo D., Tsapis N., Fattal E., Fresta M. (2015). Aqueous-Core PEG-coated PLA nanocapsules for an efficient entrapment of water soluble anticancer drugs and a smart therapeutic response. Eur. J. Pharm. Biopharm..

[18] Yun G., Hassan Z., Lee J., Kim J., Lee N.S., Kim N.H., Baek K., Hwang I., Park C.G., Kim K. (2014). Highly stable, water-dispersible metal-nanoparticle-decorated polymer nanocapsules and their catalytic applications. Angew. Chem. Int. Ed..

[19] Silva H.D., Cerqueira M.Â., Vicente A.A. (2012). Nanoemulsions for food applications: Development and characterization. Food Bioprocess Technol..

[20] Sugumar S., Nirmala J., Ghosh V., Anjali H., Mukherjee A., Chandrasekaran N. (2013). Bio-Based nanoemulsion formulation, characterization and antibacterial activity against food-borne pathogens. J. Basic Microbiol..

[21] Gupta A., Eral H.B., Hatton T.A., Doyle P.S. (2016). Nanoemulsions: Formation, properties and applications. Soft Matter.

[22] Liang J., Yan H., Wang X., Zhou Y., Gao X., Puligundla P., Wan X. (2017). Encapsulation of epigallocatechin gallate in zein/chitosan nanoparticles for controlled applications in food systems. Food Chem..

[23] Mozafari M.R., Johnson C., Hatziantoniou S., Demetzos C. (2008). Nanoliposomes and their applications in food nanotechnology. J. Liposome Res..

[24] López O.V., Castillo L.A., Garcia M.A., Villar M.A., Barbosa S.E. (2015). Food packaging bags based on thermoplastic corn starch reinforced with talc nanoparticles. Food Hydrocoll..

[25] Sutarlie L., Ow S.Y., Su X. (2017). Nanomaterials-Based biosensors for detection of microorganisms and microbial toxins. Biotechnol. J..

[26] Fuertes G., Soto I., Carrasco R., Vargas M., Sabattin J., Lagos C. (2016). Intelligent packaging systems: Sensors and nanosensors to monitor food quality and safety. J. Sens..

[27] Majdinasab M., Hayat A., Marty J.L. (2018). Aptamer-Based assays and aptasensors for detection of pathogenic bacteria in food samples. TrAC Trends Anal. Chem..

[28] Luykx D.M., Peters R.J., van Ruth S.M., Bouwmeester H. (2008). A review of analytical methods for the identification and characterization of nano delivery systems in food. J. Agric. Food Chem..

[29] Singh P. (2018). Nanotechnology in food preservation. Food Sci..

[30] Kaya-Celiker H., Mallikarjunan K. (2012). Better nutrients and therapeutics delivery in food through nanotechnology. Food Eng. Rev..

[31] Llorens A., Lloret E., Picouet P.A., Trbojevich R., Fernandez A. (2012). Metallic-Based micro and nanocomposites in food contact materials and active food packaging. Trends Food Sci. Technol..

[32] de Azeredo H.M.C., Mattoso L.H.C., McHugh T.H. (2011). Nanocomposites in food packaging–A review. Advances in Diverse Industrial Applications of Nanocomposites.

[33] Rai M., Ingle A.P., Gupta I., Pandit R., Paralikar P., Gade A., Chaud M.V., dos Santos C.A. (2019). Smart nanopackaging for the enhancement of food shelf life. Environ. Chem. Lett..

[34] Dasgupta N., Ranjan S. (2018). Nanotechnology in food sector. An Introduction to Food Grade Nanoemulsions.

[35] Yang Y., Guo Y., Sun R., Wang X. (2016). Self-Assembly and β-carotene loading capacity of hydroxyethyl cellulose-graft-linoleic acid nanomicelles. Carbohydr. Polym..

[36] Dickinson E. (2015). Microgels—An alternative colloidal ingredient for stabilization of food emulsions. Trends Food Sci. Technol..

[37] Abbas K., Saleh A., Mohamed A., MohdAzhan N. (2009). The recent advances in the nanotechnology and its applications in food processing: A review. J. Foodagric. Environ..

[38] Anjum M., Pradhan S.N. (2018). Application of nanotechnology in precision farming: A review. IJCS.

[39] Duhan J.S., Kumar R., Kumar N., Kaur P., Nehra K., Duhan S. (2017). Nanotechnology: The new perspective in precision agriculture. Biotechnol. Rep..

[40] McClements D.J., DeLoid G., Pyrgiotakis G., Shatkin J.A., Xiao H., Demokritou P. (2016). The role of the food matrix and gastrointestinal tract in the assessment of biological properties of ingested engineered nanomaterials (iENMs): State of the science and knowledge gaps. NanoImpact.

[41] Cho Y.-H., Jones O.G. (2019). Assembled protein nanoparticles in food or nutrition applications. Adv. Food Nutr. Res..

[42] Yoo S.M., Keum K.C., Yoo S.Y., Choi J.Y., Chang K.H., Yoo N.C., Yoo W.M., Kim J.M., Lee D., Lee S.Y. (2004). Development of DNA microarray for pathogen detection. Biotechnol. Bioprocess Eng..

[43] Chellaram C., Murugaboopathi G., John A., Sivakumar R., Ganesan S., Krithika S., Priya G. (2014). Significance of nanotechnology in food industry. APCBEE Procedia.

[44] Farooq S., Gull A., Ahmad T., Masoodi F., Rather S.A., Dar A.H. Emerging role of nanotechnology in food industry. Proceedings of the 5th International Conference on Nanotechnology for Better Living (NBL-2019).

[45] Espitia P.J.P., Soares N.D.F.F., dos Reis Coimbra J.S., de Andrade N.J., Cruz R.S., Medeiros E.A.A. (2012). Zinc oxide nanoparticles: Synthesis, antimicrobial activity and food packaging applications. Food Bioprocess Technol..

[46] Ezhilarasi P., Karthik P., Chhanwal N., Anandharamakrishnan C. (2013). Nanoencapsulation techniques for food bioactive components: A review. Food Bioprocess Technol..

[47] Singh T., Shukla S., Kumar P., Wahla V., Bajpai V.K., Rather I.A. (2017). Application of nanotechnology in food science: Perception and overview. Front. Microbiol..

[48] Salgado P.R., Di Giorgio L., Musso Y.S., Mauri A.N. (2019). Bioactive packaging: combining nanotechnologies with packaging for improved food functionality. Nanomaterials for Food Applications.

[49] Neethirajan S., Jayas D.S. (2011). Nanotechnology for the food and bioprocessing industries. Food Bioprocess Technol..

[50] Patra J.K., Shin H.-S., Paramithiotis S. (2018). Application of nanotechnology in food science and food microbiology. Front. Microbiol..

[51] He X., Hwang H.-M. (2016). Nanotechnology in food science: Functionality, applicability, and safety assessment. J. Food Drug Anal..

[52] Rodrigues S.M., Demokritou P., Dokoozlian N., Hendren C.O., Karn B., Mauter M.S., Sadik O.A., Safarpour M., Unrine J.M., Viers J. (2017). Nanotechnology for sustainable food production: Promising opportunities and scientific challenges. Environ. Sci. Nano.

[53] Siemer S., Hahlbrock A., Vallet C., McClements D.J., Balszuweit J., Voskuhl J., Docter D., Wessler S., Knauer S.K., Westmeier D. (2018). Nanosized food additives impact beneficial and pathogenic bacteria in the human gut: A simulated gastrointestinal study. Npj Sci. Food.

[54] Peters R.J., Bouwmeester H., Gottardo S., Amenta V., Arena M., Brandhoff P., Marvin H.J., Mech A., Moniz F.B., Pesudo L.Q. (2016). Nanomaterials for products and application in agriculture, feed and food. Trends Food Sci. Technol..

[55] Chaudhry Q., Scotter M., Blackburn J., Ross B., Boxall A., Castle L., Aitken R., Watkins R. (2008). Applications and implications of nanotechnologies for the food sector. Food Addit. Contam..

[56] Pradhan N., Singh S., Ojha N., Shrivastava A., Barla A., Rai V., Bose S. (2015). Facets of nanotechnology as seen in food processing, packaging, and preservation industry. BioMed Res. Int..

[57] Dube A., Ng K., Nicolazzo J.A., Larson I. (2010). Effective use of reducing agents and nanoparticle encapsulation in stabilizing catechins in alkaline solution. Food Chem..

[58] Jafarizadeh-Malmiri H., Sayyar Z., Anarjan N., Berenjian A. (2019). Nano-Sensors in food nanobiotechnology. Nanobiotechnology in Food: Concepts, Applications and Perspectives.

[59] Drusch S., Mannino S. (2009). Patent-Based review on industrial approaches for the microencapsulation of oils rich in polyunsaturated fatty acids. Trends Food Sci. Technol..

[60] Weiss J., Takhistov P., McClements D.J. (2006). Functional materials in food nanotechnology. J. Food Sci..

[61] Lamprecht A., Saumet J.-L., Roux J., Benoit J.-P. (2004). Lipid nanocarriers as drug delivery system for ibuprofen in pain treatment. Int. J. Pharm..

[62] Sastry R.K., Anshul S., Rao N. (2013). Nanotechnology in food processing sector–An assessment of emerging trends. J. Food Sci. Technol..

[63] Nakagawa K. (2014). Nano-and microencapsulation of flavor in food systems. Nano-and Microencapsulation for Foods.

[64] Yang R., Zhou Z., Sun G., Gao Y., Xu J., Strappe P., Blanchard C., Cheng Y., Ding X. (2015). Synthesis of homogeneous protein-stabilized rutin nanodispersions by reversible assembly of soybean (Glycine max) seed ferritin. RSC Adv..

[65] Kumar L.Y. (2015). Role and adverse effects of nanomaterials in food technology. J. Toxicol. Health.

[66] Oehlke K., Adamiuk M., Behsnilian D., Gräf V., Mayer-Miebach E., Walz E., Greiner R. (2014). Potential bioavailability enhancement of bioactive compounds using food-grade engineered nanomaterials: A review of the existing evidence. Food Funct..

[67] Haider A., Kang I.-K. (2015). Preparation of silver nanoparticles and their industrial and biomedical applications: A comprehensive review. Adv. Mater. Sci. Eng..

[68] Zhang H., Chen S. (2019). Nanoparticle-Based methods for food safety evaluation. Evaluation Technologies for Food Quality.

[69] Abraham A. (2016). Understanding the Effect of Phytochemical Coated Silver Nanoparticles on Mammalian Cells and the Protein Interactions with the Surface Corona of These Nanoparticles. Ph.D. Thesis.

[70] Arroyo B.J., Santos A.P., de Melo E.d.A., Campos A., Lins L., Boyano-Orozco L.C. (2019). Bioactive compounds and their potential use as ingredients for food and its application in food packaging. Bioactive Compounds.

[71] Clemente J.C., Pehrsson E.C., Blaser M.J., Sandhu K., Gao Z., Wang B., Magris M., Hidalgo G., Contreras M., Noya-Alarcón Ó. (2015). The microbiome of uncontacted Amerindians. Sci. Adv..

[72] Couch L.M., Wien M., Brown J.L., Davidson P. (2016). Food nanotechnology: Proposed uses, safety concerns and regulations. Agro Food Ind. Hi Tech.

[73] Abo-Elseoud W.S., Hassan M.L., Sabaa M.W., Basha M., Hassan E.A., Fadel S.M. (2018). Chitosan nanoparticles/cellulose nanocrystals nanocomposites as a carrier system for the controlled release of repaglinide. Int. J. Biol. Macromol..

[74] Fortunati E., Mazzaglia A., Balestra G.M. (2019). Sustainable control strategies for plant protection and food packaging sectors by natural substances and novel nanotechnological approaches. J. Sci. Food Agric..

[75] Ghosh C., Bera D., Roy L. (2019). Role of Nanomaterials in Food Preservation. Microbial Nanobionics.

[76] Jafarizadeh-Malmiri H., Sayyar Z., Anarjan N., Berenjian A. (2019). Nanobiotechnology in food packaging. Nanobiotechnology in Food: Concepts, Applications and Perspectives.

[77] Kuswandi B., Moradi M. (2019). Improvement of food packaging based on functional nanomaterial. Nanotechnology: Applications in Energy, Drug and Food.

[78] Rhim J.-W., Park H.-M., Ha C.-S. (2013). Bio-Nanocomposites for food packaging applications. Prog. Polym. Sci..

[79] Schaefer D., Cheung W.M. (2018). Smart packaging: Opportunities and challenges. Procedia CIRP.

[80] Zhang H. (2016). Gas Barrier Properties of Polymer Packaging: Influence on Food Shelf Life Following Microwave-Assisted Thermal Sterilization. Ph.D. Thesis.

[81] Morris V. (2011). Emerging roles of engineered nanomaterials in the food industry. Trends Biotechnol..

[82] Dinh N.X., Quy N.V., Huy T.Q., Le A.-T. (2015). Decoration of silver nanoparticles on multiwalled carbon nanotubes: Antibacterial mechanism and ultrastructural analysis. J. Nanomater..

[83] Maestri E., Marmiroli N., Song J., White J.C. (2019). Ethical issues of engineered nanomaterials applications and regulatory solutions. Exposure to Engineered Nanomaterials in the Environment.

[84] Momin J.K., Jayakumar C., Prajapati J.B. (2013). Potential of nanotechnology in functional foods. Emir. J. Food Agric..

[85] Raynes J.K., Carver J.A., Gras S.L., Gerrard J.A. (2014). Protein nanostructures in food-Should we be worried?. Trends Food Sci. Technol..

[86] Ingale A.G., Chaudhari A.N. (2018). Nanotechnology in the Food Industry. Nanotechnology, Food Security and Water Treatment.

[87] Gálvez A., Abriouel H., López R.L., Omar N.B. (2007). Bacteriocin-Based strategies for food biopreservation. Int. J. Food Microbiol..

[88] Duncan T.V. (2011). Applications of nanotechnology in food packaging and food safety: Barrier materials, antimicrobials and sensors. J. Colloid Interface Sci..

[89] Shivakumar N., Madhusudan P., Daniel S.K. (2018). Nanomaterials for smart food packaging. Handbook of Nanomaterials for Industrial Applications.

[90] Rai M., Gade A., Gaikwad S., Marcato P.D., Durán N. (2012). Biomedical applications of nanobiosensors: The state-of-the-art. J. Braz. Chem. Soc..

[91] Pathakoti K., Manubolu M., Hwang H.-M. (2017). Nanostructures: Current uses and future applications in food science. J. Food Drug Anal..

[92] Sánchez-Acevedo Z.C., Riu J., Rius F.X. (2009). Fast picomolar selective detection of bisphenol A in water using a carbon nanotube field effect transistor functionalized with estrogen receptor-α. Biosens. Bioelectron..

[93] Scandurra G., Arena A., Ciofi C., Saitta G. (2013). Electrical characterization and hydrogen peroxide sensing properties of gold/nafion: Polypyrrole/MWCNTs electrochemical devices. Sensors.

[94] Villamizar R.A., Maroto A., Rius F.X., Inza I., Figueras M.J. (2008). Fast detection of Salmonella Infantis with carbon nanotube field effect transistors. Biosens. Bioelectron..

[95] Welch C., Banks C., Simm A., Compton R. (2005). Silver nanoparticle assemblies supported on glassy-carbon electrodes for the electro-analytical detection of hydrogen peroxide. Anal. Bioanal. Chem..

[96] Thakur M., Ragavan K. (2013). Biosensors in food processing. J. Food Sci. Technol..

[97] Xiang L., Zhao C., Wang J. (2011). Nanomaterials-Based electrochemical sensors and biosensors for pesticide detection. Sens. Lett..

[98] Radoi A., Targa M., Prieto-Simon B., Marty J.-L. (2008). Enzyme-Linked immunosorbent assay (ELISA) based on superparamagnetic nanoparticles for aflatoxin M1 detection. Talanta.

[99] Sharma A., Matharu Z., Sumana G., Solanki P.R., Kim C., Malhotra B. (2010). Antibody immobilized cysteamine functionalized-gold nanoparticles for aflatoxin detection. Thin Solid Films.

[100] Xiulan S., Xiaolian Z., Jian T., Zhou J., Chu F. (2005). Preparation of gold-labeled antibody probe and its use in immunochromatography assay for detection of aflatoxin B1. Int. J. Food Microbiol..

[101] Zhang W., Tang H., Geng P., Wang Q., Jin L., Wu Z. (2007). Amperometric method for rapid detection of Escherichia coli by flow injection analysis using a bismuth nano-film modified glassy carbon electrode. Electrochem. Commun..

[102] Chiao D.-J., Shyu R.-H., Hu C.-S., Chiang H.-Y., Tang S.-S. (2004). Colloidal gold-based immunochromatographic assay for detection of botulinum neurotoxin type B. J. Chromatogr. B.

[103] Zhou Y., Pan F.-G., Li Y.-S., Zhang Y.-Y., Zhang J.-H., Lu S.-Y., Ren H.-L., Liu Z.-S. (2009). Colloidal gold probe-based immunochromatographic assay for the rapid detection of brevetoxins in fishery product samples. Biosens. Bioelectron..

[104] Wang L., Chen W., Xu D., Shim B.S., Zhu Y., Sun F., Liu L., Peng C., Jin Z., Xu C. (2009). Simple, rapid, sensitive, and versatile SWNT-paper sensor for environmental toxin detection competitive with ELISA. Nano Lett..

[105] Zamolo V.A., Valenti G., Venturelli E., Chaloin O., Marcaccio M., Boscolo S., Castagnola V., Sosa S., Berti F., Fontanive G. (2012). Highly sensitive electrochemiluminescent nanobiosensor for the detection of palytoxin. ACS Nano.

[106] Kumar S., Shukla A., Baul P.P., Mitra A., Halder D. (2018). Biodegradable hybrid nanocomposites of chitosan/gelatin and silver nanoparticles for active food packaging applications. Food Packag. Shelf Life.

[107] Ranjan S., Dasgupta N., Chakraborty A.R., Samuel S.M., Ramalingam C., Shanker R., Kumar A. (2014). Nanoscience and nanotechnologies in food industries: Opportunities and research trends. J. Nanopart. Res..

[108] Arshak K., Adley C., Moore E., Cunniffe C., Campion M., Harris J. (2007). Characterisation of polymer nanocomposite sensors for quantification of bacterial cultures. Sens. Actuators B Chem..

[109] Yang D., Zhu L., Jiang X. (2010). Electrochemical reaction mechanism and determination of Sudan I at a multi wall carbon nanotubes modified glassy carbon electrode. J. Electroanal. Chem..

[110] Fang Z., Zhao Y., Warner R.D., Johnson S.K. (2017). Active and intelligent packaging in meat industry. Trends Food Sci. Technol..

[111] Baranwal A., Mahato K., Srivastava A., Maurya P.K., Chandra P. (2016). Phytofabricated metallic nanoparticles and their clinical applications. RSC Adv..

[112] Holzinger M., Le Goff A., Cosnier S. (2014). Nanomaterials for biosensing applications: A review. Front. Chem..

[113] Kahraman M., Yazıcı M.M., Şahin F., Çulha M. (2008). Convective assembly of bacteria for surface-enhanced Raman scattering. Langmuir.

[114] Zuo P., Li X., Dominguez D.C., Ye B.-C. (2013). A PDMS/paper/glass hybrid microfluidic biochip integrated with aptamer-functionalized graphene oxide nano-biosensors for one-step multiplexed pathogen detection. Lab Chip.

[115] Chen S., Ma L., Yuan R., Chai Y., Xiang Y., Wang C. (2011). Electrochemical sensor based on Prussian blue nanorods and gold nanochains for the determination of H_2_O_2_. Eur. Food Res. Technol..

[116] Ghasemi-Varnamkhasti M., Mohtasebi S., Rodriguez-Mendez M., Siadat M., Ahmadi H., Razavi S. (2011). Electronic and bioelectronic tongues, two promising analytical tools for the quality evaluation of non alcoholic beer. Trends Food Sci. Technol..

[117] Sondi I., Salopek-Sondi B. (2004). Silver nanoparticles as antimicrobial agent: A case study on *E. coli* as a model for Gram-negative bacteria. J. Colloid Interface Sci..

[118] Wang L., Luo J., Shan S., Crew E., Yin J., Zhong C.-J., Wallek B., Wong S.S. (2011). Bacterial inactivation using silver-coated magnetic nanoparticles as functional antimicrobial agents. Anal. Chem..

[119] Thakur B., Zhou G., Chang J., Pu H., Jin B., Sui X., Yuan X., Yang C.-H., Magruder M., Chen J. (2018). Rapid detection of single *E. coli* bacteria using a graphene-based field-effect transistor device. Biosens. Bioelectron..

[120] Tominaga T. (2018). Rapid detection of *Klebsiella pneumoniae*, *Klebsiella oxytoca*, *Raoultella ornithinolytica* and other related bacteria in food by lateral-flow test strip immunoassays. J. Microbiol. Methods.

[121] Liu D., Chen W., Wei J., Li X., Wang Z., Jiang X. (2012). A highly sensitive, dual-readout assay based on gold nanoparticles for organophosphorus and carbamate pesticides. Anal. Chem..

[122] Ji X., Zheng J., Xu J., Rastogi V.K., Cheng T.-C., DeFrank J.J., Leblanc R.M. (2005). (CdSe) ZnS quantum dots and organophosphorus hydrolase bioconjugate as biosensors for detection of paraoxon. J. Phys. Chem. B.

[123] Bagheri H., Afkhami A., Shirzadmehr A., Khoshsafar H., Khoshsafar H., Ghaedi H. (2013). Novel potentiometric sensor for the determination of Cd^2+^ based on a new nano-composite. Int. J. Environ. Anal. Chem..

[124] Varshney M., Li Y., Srinivasan B., Tung S. (2007). A label-free, microfluidics and interdigitated array microelectrode-based impedance biosensor in combination with nanoparticles immunoseparation for detection of *Escherichia coli* O157: H7 in food samples. Sens. Actuators B Chem..

[125] Wang C., Irudayaraj J. (2008). Gold nanorod probes for the detection of multiple pathogens. Small.

[126] Majidi M.R., Baj R.F.B., Naseri A. (2013). Carbon nanotube-ionic liquid (CNT-IL) nanocamposite modified sol-gel derived carbon-ceramic electrode for simultaneous determination of sunset yellow and tartrazine in food samples. Food Anal. Methods.

[127] Najafi M., Khalilzadeh M.A., Karimi-Maleh H. (2014). A new strategy for determination of bisphenol A in the presence of Sudan I using a ZnO/CNTs/ionic liquid paste electrode in food samples. Food Chem..

[128] Karimi-Maleh H., Moazampour M., Yoosefian M., Sanati A.L., Tahernejad-Javazmi F., Mahani M. (2014). An electrochemical nanosensor for simultaneous voltammetric determination of ascorbic acid and Sudan I in food samples. Food Anal. Methods.

[129] Wei S., Zhao F., Xu Z., Zeng B. (2006). Voltammetric determination of folic acid with a multi-walled carbon nanotube-modified gold electrode. Microchim. Acta.

[130] Xiao F., Ruan C., Liu L., Yan R., Zhao F., Zeng B. (2008). Single-Walled carbon nanotube-ionic liquid paste electrode for the sensitive voltammetric determination of folic acid. Sens. Actuators B Chem..

[131] Vermeir S., Nicolai B., Jans K., Maes G., Lammertyn J. (2007). High-Throughput microplate enzymatic assays for fast sugar and acid quantification in apple and tomato. J. Agric. Food Chem..

[132] Verstrepen K.J., Iserentant D., Malcorps P., Derdelinckx G., Van Dijck P., Winderickx J., Pretorius I.S., Thevelein J.M., Delvaux F.R. (2004). Glucose and sucrose: Hazardous fast-food for industrial yeast?. Trends Biotechnol..

[133] Filippo E., Serra A., Manno D. (2009). Poly (vinyl alcohol) capped silver nanoparticles as localized surface plasmon resonance-based hydrogen peroxide sensor. Sens. Actuators B Chem..

[134] Liang K.-Z., Mu W.-J. (2008). ZrO_2_/DNA-derivated polyion hybrid complex membrane for the determination of hydrogen peroxide in milk. Ionics.

[135] Liu Y.-J., Yao D.-J., Chang H.-Y., Liu C.-M., Chen C. (2008). Magnetic bead-based DNA detection with multi-layers quantum dots labeling for rapid detection of *Escherichia coli* O157: H7. Biosens. Bioelectron..

[136] Ye J.-S., Wen Y., De Zhang W., Gan L.M., Xu G.Q., Sheu F.-S. (2004). Nonenzymatic glucose detection using multi-walled carbon nanotube electrodes. Electrochem. Commun..

[137] Mak A.C., Osterfeld S.J., Yu H., Wang S.X., Davis R.W., Jejelowo O.A., Pourmand N. (2010). Sensitive giant magnetoresistive-based immunoassay for multiplex mycotoxin detection. Biosens. Bioelectron..

[138] Actis P., Jejelowo O., Pourmand N. (2010). Ultrasensitive mycotoxin detection by STING sensors. Biosens. Bioelectron..

[139] Ansari A.A., Kaushik A., Solanki P.R., Malhotra B. (2010). Nanostructured zinc oxide platform for mycotoxin detection. Bioelectrochemistry.

[140] Kaushik A., Solanki P.R., Ansari A.A., Ahmad S., Malhotra B.D. (2009). A nanostructured cerium oxide film-based immunosensor for mycotoxin detection. Nanotechnology.

[141] Norouzi P., Larijani B., Ganjali M. (2012). Ochratoxin A sensor based on nanocomposite hybrid film of ionic liquid-graphene nano-sheets using coulometric FFT cyclic voltammetry. Int. J. Electrochem. Sci..

[142] Zhang C., Yin A.-X., Jiang R., Rong J., Dong L., Zhao T., Sun L.-D., Wang J., Chen X., Yan C.-H. (2013). Time-Temperature indicator for perishable products based on kinetically programmable Ag overgrowth on Au nanorods. ACS Nano.

[143] Zhao X., Hilliard L.R., Mechery S.J., Wang Y., Bagwe R.P., Jin S., Tan W. (2004). A rapid bioassay for single bacterial cell quantitation using bioconjugated nanoparticles. Proc. Natl. Acad. Sci. USA.

[144] Li Y., Cu Y.T.H., Luo D. (2005). Multiplexed detection of pathogen DNA with DNA-based fluorescence nanobarcodes. Nat. Biotechnol..

[145] Su X.-L., Li Y. (2004). Quantum dot biolabeling coupled with immunomagnetic separation for detection of *Escherichia coli* O157: H7. Anal. Chem..

[146] García-Aljaro C., Bangar M.A., Baldrich E., Muñoz F.J., Mulchandani A. (2010). Conducting polymer nanowire-based chemiresistive biosensor for the detection of bacterial spores. Biosens. Bioelectron..

[147] Afonso A.S., Pérez-López B., Faria R.C., Mattoso L.H., Hernández-Herrero M., Roig-Sagués A.X., Maltez-da Costa M., Merkoçi A. (2013). Electrochemical detection of Salmonella using gold nanoparticles. Biosens. Bioelectron..

[148] Joung H.-A., Lee N.-R., Lee S.K., Ahn J., Shin Y.B., Choi H.-S., Lee C.-S., Kim S., Kim M.-G. (2008). High sensitivity detection of 16s rRNA using peptide nucleic acid probes and a surface plasmon resonance biosensor. Anal. Chim. Acta.

[149] Kumar V., Guleria P., Mehta S.K. (2017). Nanosensors for food quality and safety assessment. Environ. Chem. Lett..

[150] Zhao G., Xing F., Deng S. (2007). A disposable amperometric enzyme immunosensor for rapid detection of Vibrio parahaemolyticus in food based on agarose/nano-Au membrane and screen-printed electrode. Electrochem. Commun..

[151] Amagliani G., Brandi G., Omiccioli E., Casiere A., Bruce I.J., Magnani M. (2004). Direct detection of Listeria monocytogenes from milk by magnetic based DNA isolation and PCR. Food Microbiol..

[152] Cheng Y., Liu Y., Huang J., Li K., Zhang W., Xian Y., Jin L. (2009). Combining biofunctional magnetic nanoparticles and ATP bioluminescence for rapid detection of *Escherichia coli*. Talanta.

[153] Zhou Z., Li T., Huang H., Chen Y., Liu F., Huang C., Li N. (2014). A dual amplification strategy for DNA detection combining bio-barcode assay and metal-enhanced fluorescence modality. Chem. Commun..

[154] Dungchai W., Siangproh W., Chaicumpa W., Tongtawe P., Chailapakul O. (2008). *Salmonella typhi* determination using voltammetric amplification of nanoparticles: A highly sensitive strategy for metalloimmunoassay based on a copper-enhanced gold label. Talanta.

[155] Fu J., Park B., Siragusa G., Jones L., Tripp R., Zhao Y., Cho Y.-J. (2008). An Au/Si hetero-nanorod-based biosensor for Salmonella detection. Nanotechnology.

[156] De Souza Rebouças J., Esparza I., Ferrer M., Sanz M.L., Irache J.M., Gamazo C. (2012). Nanoparticulate adjuvants and delivery systems for allergen immunotherapy. Biomed Res. Int..

[157] Oh S.Y., Heo N.S., Shukla S., Cho H.-J., Vilian A.E., Kim J., Lee S.Y., Han Y.-K., Yoo S.M., Huh Y.S. (2017). Development of gold nanoparticle-aptamer-based LSPR sensing chips for the rapid detection of *Salmonella typhimurium* in pork meat. Sci. Rep..

[158] Hahn M.A., Keng P.C., Krauss T.D. (2008). Flow cytometric analysis to detect pathogens in bacterial cell mixtures using semiconductor quantum dots. Anal. Chem..

[159] Tully E., Hearty S., Leonard P., O’Kennedy R. (2006). The development of rapid fluorescence-based immunoassays, using quantum dot-labelled antibodies for the detection of *Listeria monocytogenes* cell surface proteins. Int. J. Biol. Macromol..

[160] Yang L., Li Y. (2005). Quantum dots as fluorescent labels for quantitative detection of *Salmonella typhimurium* in chicken carcass wash water. J. Food Prot..

[161] Zhao Y., Ye M., Chao Q., Jia N., Ge Y., Shen H. (2008). Simultaneous detection of multifood-borne pathogenic bacteria based on functionalized quantum dots coupled with immunomagnetic separation in food samples. J. Agric. Food Chem..

[162] Shukla S., Lee G., Song X., Park S., Kim M. (2016). Immunoliposome-Based immunomagnetic concentration and separation assay for rapid detection of *Cronobacter sakazakii*. Biosens. Bioelectron..

[163] Zhou L., Lv S., He G., He Q., Shi B. (2011). Effect of PE/Ag_2_O nano-packaging on the quality of apple slices. J. Food Qual..

[164] Kara M., Uzun L., Kolayli S., Denizli A. (2013). Combining molecular imprinted nanoparticles with surface plasmon resonance nanosensor for chloramphenicol detection in honey. J. Appl. Polym. Sci..

[165] Gan T., Li K., Wu K. (2008). Multi-Wall carbon nanotube-based electrochemical sensor for sensitive determination of Sudan I. Sens. Actuators B Chem..

[166] Wu M., Tang W., Guimarães J., Wang Q., He P., Fang Y. (2013). Electrochemical detection of Sudan I using a multi-walled carbon nanotube/chitosan composite modified glassy carbon electrode. Am. J. Anal. Chem..

[167] Yin H., Zhou Y., Meng X., Tang T., Ai S., Zhu L. (2011). Electrochemical behaviour of Sudan I at Fe_3_O_4_ nanoparticles modified glassy carbon electrode and its determination in food samples. Food Chem..

[168] Constantine C.A., Gattás-Asfura K.M., Mello S.V., Crespo G., Rastogi V., Cheng T.-C., DeFrank J.J., Leblanc R.M. (2003). Layer-by-layer films of chitosan, organophosphorus hydrolase and thioglycolic acid-capped CdSe quantum dots for the detection of paraoxon. J. Phys. Chem. B.

[169] Vamvakaki V., Chaniotakis N.A. (2007). Pesticide detection with a liposome-based nano-biosensor. Biosens. Bioelectron..

[170] Wang M., Li Z. (2008). Nano-Composite ZrO_2_/Au film electrode for voltammetric detection of parathion. Sens. Actuators B Chem..

[171] Devaramani S., Malingappa P. (2012). Synthesis and characterization of cobalt nitroprusside nano particles: Application to sulfite sensing in food and water samples. Electrochim. Acta.

[172] Han C., Li H. (2010). Visual detection of melamine in infant formula at 0.1 ppm level based on silver nanoparticles. Analyst.

[173] Huang H., Li L., Zhou G., Liu Z., Ma Q., Feng Y., Zeng G., Tinnefeld P., He Z. (2011). Visual detection of melamine in milk samples based on label-free and labeled gold nanoparticles. Talanta.

[174] Kuang H., Chen W., Yan W., Xu L., Zhu Y., Liu L., Chu H., Peng C., Wang L., Kotov N.A. (2011). Crown ether assembly of gold nanoparticles: Melamine sensor. Biosens. Bioelectron..

[175] Ping H., Zhang M., Li H., Li S., Chen Q., Sun C., Zhang T. (2012). Visual detection of melamine in raw milk by label-free silver nanoparticles. Food Control.

[176] Su H., Fan H., Ai S., Wu N., Fan H., Bian P., Liu J. (2011). Selective determination of melamine in milk samples using 3-mercapto-1-propanesulfonate-modified gold nanoparticles as colorimetric probe. Talanta.

[177] Ensafi A.A., Karimi-Maleh H., Mallakpour S. (2012). Simultaneous determination of Ascorbic Acid, Acetaminophen, and Tryptophan by square wave voltammetry using N-(3,4-Dihydroxyphenethyl)-3,5-Dinitrobenzamide-Modified Carbon Nanotubes paste electrode. Electroanalysis.

[178] Wang S., Zhang Q., Wang R., Yoon S. (2003). A novel multi-walled carbon nanotube-based biosensor for glucose detection. Biochem. Biophys. Res. Commun..

[179] Yemini M., Reches M., Gazit E., Rishpon J. (2005). Peptide nanotube-modified electrodes for enzyme-biosensor applications. Anal. Chem..

[180] Beitollahi H., Ardakani M.M., Ganjipour B., Naeimi H. (2008). Novel 2,2′-[1,2-ethanediylbis (nitriloethylidyne)]-bis-hydroquinone double-wall carbon nanotube paste electrode for simultaneous determination of epinephrine, uric acid and folic acid. Biosens. Bioelectron..

[181] Jamali T., Karimi-Maleh H., Khalilzadeh M.A. (2014). A novel nanosensor based on Pt: Co nanoalloy ionic liquid carbon paste electrode for voltammetric determination of vitamin B9 in food samples. LWT Food Sci. Technol..

[182] Wu R.-J., Lin D.-J., Yu M.-R., Chen M.H., Lai H.-F. (2013). Ag@SnO_2_ core-shell material for use in fast-response ethanol sensor at room operating temperature. Sens. Actuators B Chem..

[183] Liu S., Yu J., Ju H. (2003). Renewable phenol biosensor based on a tyrosinase-colloidal gold modified carbon paste electrode. J. Electroanal. Chem..

[184] Sanz V.C., Mena M.L., González-Cortés A., Yanez-Sedeno P., Pingarrón J. (2005). Development of a tyrosinase biosensor based on gold nanoparticles-modified glassy carbon electrodes: Application to the measurement of a bioelectrochemical polyphenols index in wines. Anal. Chim. Acta.

[185] Monteiro C.A., Cannon G., Moubarac J.-C., Levy R.B., Louzada M.L.C., Jaime P.C. (2018). The UN Decade of Nutrition, the NOVA food classification and the trouble with ultra-processing. Public Health Nutr..

[186] Rahman N.A. (2019). Applications of polymeric nanoparticles in food sector. Nanotechnology: Applications in Energy, Drug and Food.

[187] Tran Q.H., Le A.-T. (2013). Silver nanoparticles: Synthesis, properties, toxicology, applications and perspectives. Adv. Nat. Sci. Nanosci. Nanotechnol..

[188] Verma P., Maheshwari S.K. (2019). Applications of Silver nanoparticles in diverse sectors. Int. J. Nano Dimens..

[189] Sastry R.K., Rashmi H., Rao N. (2011). Nanotechnology for enhancing food security in India. Food Policy.

[190] Wesley S.J., Raja P., Raj A., Tiroutchelvamae D. (2014). Review on-nanotechnology applications in food packaging and safety. Int. J. Eng. Res..

[191] Boverhof D.R., Bramante C.M., Butala J.H., Clancy S.F., Lafranconi M., West J., Gordon S.C. (2015). Comparative assessment of nanomaterial definitions and safety evaluation considerations. Regul. Toxicol. Pharmacol..

[192] Brody A.L., Bugusu B., Han J.H., Sand C.K., Mchugh T.H. (2008). Innovative food packaging solutions. J. Food Sci..

[193] Jung I., Ih S., Yoo H., Hong S., Park S. (2018). Fourier transform surface plasmon resonance of nanodisks embedded in magnetic nanorods. Nano Lett..

[194] Wang M., Duong B., Fenniri H., Su M. (2015). Nanomaterial-Based barcodes. Nanoscale.

[195] Qin L., Banholzer M.J., Millstone J.E., Mirkin C.A. (2007). Nanodisk codes. Nano Lett..

[196] Banu S., Birtwell S., Chen Y., Galitonov G., Morgan H., Zheludev N. (2006). High Capacity Nano-Optical Diffraction Barcode Tagging for Biological and Chemical Applications.

[197] Birtwell S., Galitonov G., Morgan H., Zheludev N. (2008). Superimposed nanostructured diffraction gratings as high capacity barcodes for biological and chemical applications. Opt. Commun..

[198] Li X., Wang T., Zhang J., Zhu D., Zhang X., Ning Y., Zhang H., Yang B. (2010). Controlled fabrication of fluorescent barcode nanorods. ACS Nano.

[199] Lin C., Jungmann R., Leifer A.M., Li C., Levner D., Church G.M., Shih W.M., Yin P. (2012). Submicrometre geometrically encoded fluorescent barcodes self-assembled from DNA. Nat. Chem..

[200] Krishna V.D., Wu K., Su D., Cheeran M.C., Wang J.-P., Perez A. (2018). Nanotechnology: Review of concepts and potential application of sensing platforms in food safety. Food Microbiol..

[201] Kumar S., Dilbaghi N., Barnela M., Bhanjana G., Kumar R. (2012). Biosensors as novel platforms for detection of food pathogens and allergens. BioNanoScience.

[202] Pilolli R., Monaci L., Visconti A. (2013). Advances in biosensor development based on integrating nanotechnology and applied to food-allergen management. TrAC Trends Anal. Chem..

[203] Vogelbruch M., Nuss B., Körner M., Kapp A., Kiehl P., Bohm W. (2000). Aluminium-Induced granulomas after inaccurate intradermal hyposensitization injections of aluminium-adsorbed depot preparations. Allergy.

[204] Gamazo C., Gastaminza G., Ferrer M., Sanz M.L., Irache J.M. (2014). Nanoparticle based-immunotherapy against allergy. Immunotherapy.

[205] Pali-Schöll I., Szöllösi H., Starkl P., Scheicher B., Stremnitzer C., Hofmeister A., Roth-Walter F., Lukschal A., Diesner S.C., Zimmer A. (2013). Protamine nanoparticles with CpG-oligodeoxynucleotide prevent an allergen-induced Th2-response in BALB/c mice. Eur. J. Pharm. Biopharm..

[206] Lee B., Park J.-H., Byun J.-Y., Kim J.H., Kim M.-G. (2018). An optical fiber-based LSPR aptasensor for simple and rapid In-Situ detection of ochratoxin A. Biosens. Bioelectron..

[207] Zhang Y., Wu Q., Sun M., Zhang J., Mo S., Wang J., Wei X., Bai J. (2018). Magnetic-Assisted aptamer-based fluorescent assay for allergen detection in food matrix. Sens. Actuators B Chem..

[208] Brotons-Canto A., Gamazo C., Martín-Arbella N., Abdulkarim M., Matías J., Gumbleton M., Irache J.M. (2018). Evaluation of nanoparticles as oral vehicles for immunotherapy against experimental peanut allergy. Int. J. Biol. Macromol..

[209] Prasad A., Mahato K., Chandra P., Srivastava A., Joshi S.N., Maurya P.K. (2016). Bioinspired composite materials: Applications in diagnostics and therapeutics. J. Mol. Eng. Mater..

[210] Forier K., Raemdonck K., De Smedt S.C., Demeester J., Coenye T., Braeckmans K. (2014). Lipid and polymer nanoparticles for drug delivery to bacterial biofilms. J. Control. Release.

[211] Shakeri S., Kermanshahi R.K., Moghaddam M.M., Emtiazi G. (2007). Assessment of biofilm cell removal and killing and biocide efficacy using the microtiter plate test. Biofouling.

[212] Zhang L., Luo J., Menkhaus T.J., Varadaraju H., Sun Y., Fong H. (2011). Antimicrobial nano-fibrous membranes developed from electrospun polyacrylonitrile nanofibers. J. Membr. Sci..

[213] Gambino M., Ahmed M.A.-A.A., Villa F., Cappitelli F. (2017). Zinc oxide nanoparticles hinder fungal biofilm development in an ancient Egyptian tomb. Int. Biodeterior. Biodegrad..

[214] Saleem S., Ahmed B., Khan M.S., Al-Shaeri M., Musarrat J. (2017). Inhibition of growth and biofilm formation of clinical bacterial isolates by NiO nanoparticles synthesized from *Eucalyptus globulus* plants. Microb. Pathog..

[215] Shahrokh S., Emtiazi G. (2009). Toxicity and unusual biological behavior of nanosilver on gram positive and negative bacteria assayed by microtiter-plate. Eur. J. Biol. Sci..

[216] Ranmadugala D., Ebrahiminezhad A., Manley-Harris M., Ghasemi Y., Berenjian A. (2017). The effect of iron oxide nanoparticles on *Bacillus subtilis* biofilm, growth and viability. Process Biochem..

[217] Ahmed A., Khan A.K., Anwar A., Ali S.A., Shah M.R. (2016). Biofilm inhibitory effect of chlorhexidine conjugated gold nanoparticles against Klebsiella pneumoniae. Microb. Pathog..

[218] Vetter S.M., Schlievert P.M. (2005). Glycerol monolaurate inhibits virulence factor production in *Bacillus anthracis*. Antimicrob. Agents Chemother..

[219] Gonzalez N., Johnston L. (2018). Safety of engineered nanomaterials. Chem. Int..

[220] Yao S., Swetha P., Zhu Y. (2018). Nanomaterial-Enabled wearable sensors for healthcare. Adv. Healthc. Mater..

[221] Higashisaka K., Yoshioka Y., Tsutsumi Y. (2015). Applications and safety of nanomaterials used in the food industry. Food Saf..

[222] Chaturvedi S., Dave P.N. (2018). Nanomaterials: Environmental, human health risk. Handbook of Nanomaterials for Industrial Applications.

[223] Yuan X., Zhang X., Sun L., Wei Y., Wei X. (2019). Cellular toxicity and immunological effects of carbon-based nanomaterials. Part. Fibre Toxicol..

[224] Sing T., Shukla S., Kumar P., Wahla V., Bajpai V.K., Rather I.A. (2017). Corrigendum: Application of nanotechnology in food science: Perception and overview. Front. Microbiol..

[225] Bradley E.L., Castle L., Chaudhry Q. (2011). Applications of nanomaterials in food packaging with a consideration of opportunities for developing countries. Trends Food Sci. Technol..

[226] Athinarayanan J., Periasamy V.S., Alsaif M.A., Al-Warthan A.A., Alshatwi A.A. (2014). Presence of nanosilica (E551) in commercial food products: TNF-mediated oxidative stress and altered cell cycle progression in human lung fibroblast cells. Cell Biol. Toxicol..

[227] Mahler G.J., Esch M.B., Tako E., Southard T.L., Archer S.D., Glahn R.P., Shuler M.L. (2012). Oral exposure to polystyrene nanoparticles affects iron absorption. Nat. Nanotechnol..

[228] Karlsson H.L., Toprak M.S., Fadeel B. (2015). Toxicity of metal and metal oxide nanoparticles. Handbook on the Toxicology of Metals.

[229] Yang L., Luo X.-B., Luo S.-L. (2019). Assessment on Toxicity of Nanomaterials. Nanomaterials for the Removal of Pollutants and Resource Reutilization.

[230] Rizwan M., Ali S., Qayyum M.F., Ok Y.S., Adrees M., Ibrahim M., Zia-ur-Rehman M., Farid M., Abbas F. (2017). Effect of metal and metal oxide nanoparticles on growth and physiology of globally important food crops: A critical review. J. Hazard. Mater..

[231] Wen K.P., Chen Y.C., Chuang C.H., Chang H.Y., Lee C.Y., Tai N.H. (2015). Accumulation and toxicity of intravenously-injected functionalized graphene oxide in mice. J. Appl. Toxicol..

[232] Jeevanandam J., San Chan Y., Danquah M.K. (2019). Zebrafish as a model organism to study nanomaterial toxicity. Emerg. Sci. J..

[233] d’Amora M., Cassano D., Pocoví-Martínez S., Giordani S., Voliani V. (2018). Biodistribution and biocompatibility of passion fruit-like nano-architectures in zebrafish. Nanotoxicology.

[234] Shubha P., Gowda M.L., Namratha K., Manjunatha H., Byrappa K. (2019). In Vitro and In Vivo evaluation of green-hydrothermal synthesized ZnO nanoparticles. J. Drug Deliv. Sci. Technol..

[235] Cassano D., Mapanao A.-K., Summa M., Vlamidis Y., Giannone G., Santi M., Guzzolino E., Pitto L., Poliseno L., Bertorelli R. (2019). Biosafety and biokinetics of noble metals: The impact of their chemical nature. ACS Appl. Bio Mater..

[236] Amenta V., Aschberger K., Arena M., Bouwmeester H., Moniz F.B., Brandhoff P., Gottardo S., Marvin H.J., Mech A., Pesudo L.Q. (2015). Regulatory aspects of nanotechnology in the agri/feed/food sector in EU and non-EU countries. Regul. Toxicol. Pharmacol..

[237] Kookana R.S., Boxall A.B., Reeves P.T., Ashauer R., Beulke S., Chaudhry Q., Cornelis G., Fernandes T.F., Gan J., Kah M. (2014). Nanopesticides: Guiding principles for regulatory evaluation of environmental risks. J. Agric. Food Chem..

